# Secondary malaria vectors in western Kenya include novel species with unexpectedly high densities and parasite infection rates

**DOI:** 10.1186/s13071-021-04748-9

**Published:** 2021-05-12

**Authors:** Amine M. Mustapha, Susan Musembi, Anthony K. Nyamache, Maxwell G. Machani, Jackline Kosgei, Lucy Wamuyu, Eric Ochomo, Neil F. Lobo

**Affiliations:** 1 Department of Biochemistry and Biotechnology, Kenyatta University, P.O. Box 43844, Nairobi, Kenya; 2 Entomology Section, Centre for Global Health Research, KEMRI_KISUMU, P.O. Box 1578-40100, Kisumu, Kenya; 3 Institute of Biotechnology Research (IBR), Jomo Kenyatta University of Agriculture and Technology (JKUAT), P.O. Box 62000-00200, Nairobi, Kenya; 4 Eck Institute for Global Health, University of Notre Dame, Notre Dame, IN 46556 USA

**Keywords:** *Anopheles*, Malaria, Secondary vector, Insecticide resistance, Sporozoite infection

## Abstract

**Background:**

Malaria vector control has been implemented chiefly through indoor interventions targeting primary vectors resulting in population declines—pointing to a possible greater proportional contribution to transmission by secondary malaria vectors with their predominant exophagic and exophilic traits. With a historical focus on primary vectors, there is paucity of data on secondary malaria vectors in many countries in Africa. This study sought to determine the species compositions and bionomic traits, including proportions infected with *Plasmodium falciparum* and phenotypic insecticide resistance, of secondary vectors in three sites with high malaria transmission in Kisumu County, western Kenya.

**Methods:**

Cross-sectional sampling of adult *Anopheles* was conducted using indoor and outdoor CDC light traps (CDC-LT) and animal-baited traps (ABTs) in Kakola-Ombaka and Kisian, while larvae were sampled in Ahero. Secondary vectors captured were exposed to permethrin using WHO bioassays and then analyzed by ELISA to test for proportions infected with *P. falciparum* sporozoites. All *Anopheles* were identified to species using morphological keys with a subset being molecularly identified using ITS2 and *CO1* sequencing for species identification.

**Results:**

Two morphologically identified secondary vectors captured—*An. coustani* and *An. pharoensis*—were determined to consist of four species molecularly. These included *An. christyi*, *An. sp. 15 BSL-2014,* an unidentified member of the *An. coustani* complex (*An. cf. coustani*) and a species similar to that of *An. pharoensis* and *An. squamosus* (*An. cf. pharoensis*). Standardized (*Anopheles* per trap per night) capture rates demonstrate higher proportions of secondary vectors across most trapping methods—with overall indoor and outdoor CDC-LTs and ABT captures composed of 52.2% (*n* = 93), 78.9% (*n* = 221) and 58.1% (*n* = 573) secondary vectors respectively. Secondary vectors were primarily caught outdoors. The overall proportion of secondary vectors with *P. falciparum* sporozoite was 0.63% (*n* = 5), with the unidentified species *An. cf. pharoensis,* determined to carry *Plasmodium*. Overall secondary vectors were susceptible to permethrin with a > 99% mortality rate.

**Conclusions:**

Given their high densities, endophily equivalent to primary vectors, higher exophily and *Plasmodium*-positive proportions, secondary vectors may contribute substantially to malaria transmission. Unidentified species demonstrate the need for further morphological and molecular identification studies towards further characterization. Continued monitoring is essential for understanding their temporal contributions to transmission, the possible elevation of some to primary vectors and the development of insecticide resistance.

**Graphic Abstract:**

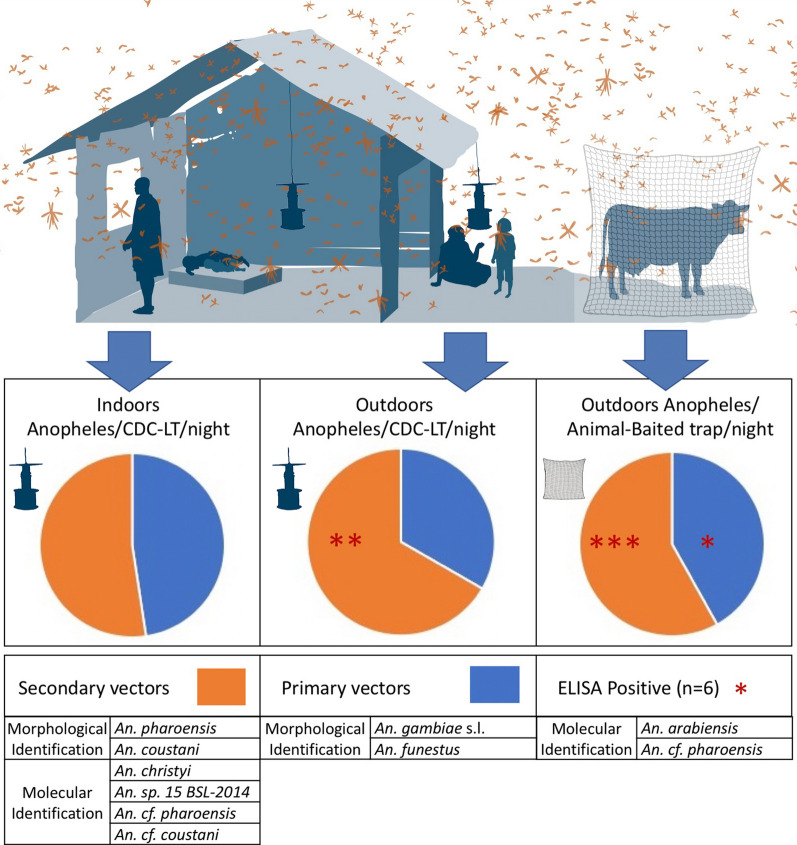

## Introduction

Malaria continues to be a burden in the sub-Saharan African region. The World Health Organization (WHO) African region recorded an estimated 215 million malaria cases and 384,000 deaths in 2019, accounting for 94% of the global burden [1]. Approximately 75% of the population in Kenya is at risk of the disease and 16% of outpatient consultations are malaria related [2]. Disease transmission in the country is variable with regions being endemic, epidemic-prone, seasonal transmission, or low risk zones, with prevalence rates of *Plasmodium falciparum* as high as 36.5% in parts of western Kenya [2, 3].

Primary vectors, described based on their abundance and quantifiable sporozoite rates, are those that are major drivers of malaria incidence [4, 5]. Primary malaria vectors in sub-Saharan Africa are predominantly anthropophilic and anthropophagic (prefer human habitation and biting humans) [4], endophilic (indoor resting) and endophagic (indoor biting) [6]. Consequently, indoor interventions such as Indoor Residual Spraying (IRS) and Long-Lasting Insecticide-Treated Nets (LLINs) are most effective against these primary vectors [7] as their behaviors overlap with where and how these interventions function.

Primary malaria vectors documented in western Kenya include *An. gambiae * (*s.s.*), *An. arabiensis* and *An. funestus* [8]. These vectors are found across Kenya with *An. merus* also being documented as a significant contributor to transmission in specific areas [9, 10]. Degefa et al. [7] demonstrated that from 2015 to 2016, 71.4% and 12.3% of *Anopheles* collected in western Kenya using multiple sampling methods were *An. gambiae* (*s.l.*) and *An. funestus*, respectively*.* Molecular analysis demonstrated that the majority of *An. gambiae* (*s.l.*) in Ahero were composed of *An. arabiensis* (98.9%) while those in nearby Iguhu were 13% with the rest being *An. gambiae* (*s.s.*). In Ahero, Kisumu County, *An. gambiae* (*s.s.*) sampled from indoor and outdoor CDC light traps had sporozoite rates of 0.38 and 0.35%, respectively, while *An. arabiensis* had a sporozoite rate of 0.16%*. An. funestus* collected from indoor CDC light traps and pyrethroid spray catches (PSCs) had sporozoite rates of 2.6 and 2.0%, respectively, suggesting a primary vector status.

Secondary vectors across Africa include species such as *An. coustani, An. ziemanni*, *An. pharoensis, An. rivulorum* and *An. squamosus* [11, 12]. Secondary vectors are those that have historically been documented as playing a minor role—contributing to an estimated 5% of malaria transmission across Africa [5]. Documented secondary malaria vectors in Ahero, Western Kenya, include *An. coustani* and *An. pharoensis* with circumsporozoite (CS) protein found in *An. coustani* specimens demonstrating the ability to transmit malaria [9]. *Anopheles ziemanni* (part of the *An. coustani* group) has also been demonstrated to be infectious with malaria in Cameroun and Rwanda [13, 14]. Since the *An. coustani* group consists of several documented species, and morphological identifications are usually used when characterizing vectors, there remains the possibility of other complex members as well as novel species being vectors from this complex [15]. Studies have documented that *An. pharoensis* is a vector in other parts of Kenya as well as Tanzania [7, 16]. Approximately 15.7% of the total *Anopheles* mosquitos collected in Mwea rice fields in Central Kenya were found to be this species with *Plasmodium* infection rates as high as 1.3% by ELISA [17]. Vectors documented with sporozoites in Zambia include *An. theileri, An. coustani* and *An. rivulorum* [18].

Most secondary vectors have historically demonstrated a preference for biting and resting outdoors [5, 15] with associated zoophily and zoophagy and may sustain malaria transmission outside the protection of indoor interventions [2, 19–21]. Though believed to be of insignificant status in malaria transmission due to their presumed zoophilic behavior, the *An. coustani* complex and *An. squamosus* have been documented to be significantly anthropophilic in Zambia—suggesting a possible greater contribution to malaria transmission [11]. In western Kenya studies have also pointed to endophily in members of the *An. coustani* complex [22].

Several non-primary *Anopheles* species have been documented in the western Kenya highlands including several novel species [7]. Known species include *An. pretoriensis An. maculipalpis, An. coustani, An. theileri, An. rufipes, An. leesoni, An. christyi* and *An. squamosus* [23]. Molecular analysis using the internal transcribed spacer region 2 (ITS2) and cytochrome oxidase subunit 1 (*CO1*) loci demonstrated the presence of novel and unknown species across multiple geographies [15, 18, 23–25]. The lack of molecular data that may distinguish species along with the reliance on historical morphological keys may overlook the presence and contributions of some species to malaria transmission.

The use of indoor-based LLINs and IRS has resulted in a significant decrease in malaria [26, 27]. Three primary selective impacts an intervention may have on vector populations include changes in vector species, changes in vector behaviors and the development and spread of insecticide resistance. Since LLINs and IRS target endophilic and endophagic vectors, primary effects would be seen on primary vectors that demonstrate the susceptible traits of endophagy and endophily. Studies have demonstrated that interventions may result in temporal changes in species composition with associated shifts in primary vector densities [28–30]. In western Kenya, extensive LLIN use resulted in relative densities of endophagic and endophilic *An. gambiae* (*s.s.*) declining while those of *An. arabiensis* increased significantly [28]. This change in species compositions may be attributed to LLIN-based mortality impacting the predominantly endophilic and endophagic *An. gambiae* (*s.s.*) relative to that of the exophagic and exophilic *An. Arabiensis*—a paradigm also reflected in other datasets [28, 30–32]. Changes in behaviors and species compositions following intervention deployment have been demonstrated in several other studies across multiple geographies [6, 19, 24, 26, 33, 34].

The development and spread of insecticide resistance have been associated with intervention implementation. Studies have associated LLIN and IRS with insecticide resistance in several malaria vectors [35–40]. For instance, extensive LLIN coverage led to an initial decrease in malaria vector density in western Kenya (Iguhu, Marani and Kombewa) between 2002 and 2007 followed by a 5–10× increase associated with the development of insecticide resistance [35]. Though insecticide resistance has been documented in primary vectors [41–43], secondary vectors are either not sampled or tested for characterizing insecticide susceptibility.

The historical focus of data collection and transmission characterization based on ‘primary’ vectors may result in a biased dataset—especially in the context of intervention-based impacts on susceptible primary vectors. There remains a lack of data on species composition, population dynamics, bionomics, insecticide resistance status and infection rates. In addition, the continued use of indoor interventions and consequent selective pressures on primary vectors may have differential impacts on secondary vectors that have different bionomic traits. The exophagic and exophilic nature of many secondary vectors allows them to circumvent the increased mortality associated with indoor interventions, with smaller effects on primary epidemiological drivers of transmission—population size and age structure. This may elevate and enhance transmission as well as undermine current efforts to eliminate malaria since they are often sympatric with primary vectors. As long as secondary vectors are able to sustain residual transmission, unchecked by predominant indoor control mechanism, malaria elimination will continue to be a major challenge. This study seeks to fill this knowledge gap by characterizing the species composition of secondary vectors, their bionomic traits, proportions infected with sporozoites and insecticide resistance frequencies to permethrin in Kisumu County, western Kenya.

## Methods

**Fig. 1:**
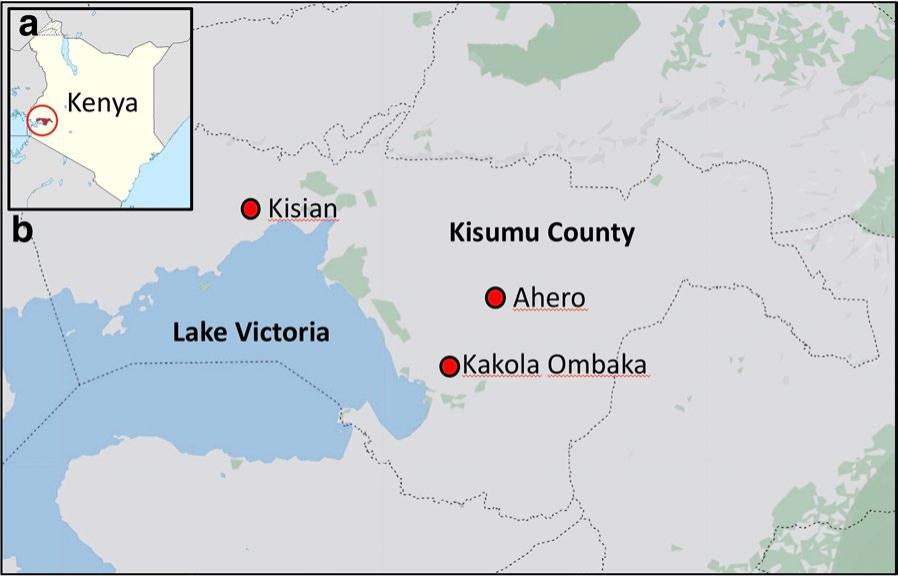
**a** The location of Kisumu County (circled) in western Kenya. **b** The location of the sampling sites in Kisumu County—Kisian, Ahero and Kakola-Ombaka

Study site: This study was conducted within three regions in Kisumu County, western Kenya—namely Kisian (00.02464°S, 033.60187°E, altitude 1280–1330 m above sea level [masl]), Ahero (00.17259° S,034.91983° E, altitude 1162–1360 masl) and Kakola-Ombaka (0.2496° S, 34.8790° E, 1142.00 m/3746.72 masl) (Fig. 1). Malaria transmission occurs throughout the year in western Kenya, with peaks corresponding to rainfall in mid-April to July and November to December. It is classified as a lake endemic region with a *Plasmodium falciparum* prevalence of 20%-50% [3]. *Anopheles gambiae (s.s.), An. arabiensis* and *An. funestus (s.s.)* are the historical primary vectors in the region [28, 44, 45]. Secondary vectors include *An. rivulorum*, *An. coustani (s.l.)* and *An. pharoensis* [5, 7, 15]. While Ahero is characterized by large irrigation fields which provide favorable larval sites for malaria vector proliferation, Kisian is known for its cattle farming which provides vectors with a source of bovine blood and brings them into increased contact with humans. Frequent flooding in lowland Kakola-Ombaka predisposes this site to vector proliferation.

### Mosquito sampling

To establish the composition and temporal density of secondary malaria vectors, and to determine their susceptibility to the pyrethroid insecticide permethrin, *Anopheles* mosquitoes were sampled in a cross-sectional study design using CDC light traps (CDC-LTs), animal (cow)-baited tent traps (ABTs) and larval dipping over the rainy season.

Paired indoor and outdoor CDC-LT catches were conducted in Kakola-Ombaka for 14 days per month for 3 months (April to June, 2019), in 20 sentinel houses per night, for a total of 840 indoor and outdoor trapping nights each. With the aim of using houses with larger odor sources, houses with more occupants (> 4 people) were selected. Indoor CDC-LTs were hung 15 cm from occupied beds, while outdoor traps were placed 1 to 2 m from windows and at a height of 4 m from the ground. Sampling started at 1900 h, and the traps were collected at 0500 h.

ABTs, using a 2–3-year-old cow, were conducted in both Kakola-Ombaka and Kisian for 14 days per month for 3 months (April to June 2019) each, using two ABTs per night per site for a total of 84 ABT trapping nights per site. ABTs were placed 3 m from the nearest house.

Larval sampling for insecticide resistance tests were conducted in Ahero for 14 days each month (April to June 2019). Collections were conducted using standard 350-ml dippers between 0900 and 1200 h per day in all identified water bodies, including ponds, swamps, marshes, irrigation water, stagnant drainage ditches and flood plains.

### Adult processing

Adult mosquitoes were morphologically identified with the help of a dissecting microscope according to standard taxonomic keys [46] and sorted according to their abdominal status. Each specimen was morphologically identified, assigned a unique code to capture the collected site, house number, date of collection and collection method and stored in 1.5-ml Eppendorf tubes on silica gel for further processing.

### Larval rearing

Collected larvae were labeled by habitat type and transported to the Entomology section, KEMRI-CGHR laboratory, fed with fish food and reared to adulthood using standardized laboratory methods [47]. Adult mosquitoes were fed on a 10% sucrose solution. Temperature was maintained at 27–30 °C and humidity at 80–85%.

### WHO insecticide susceptibility assay

Insecticide susceptibility tests were conducted on both adults captured as well as larvae raised to adults. Adults captured (all females) were immediately transported to the laboratory and sugar fed. Insecticide susceptibility tests using 0.75% permethrin-impregnated papers, following standard insecticide susceptibility monitoring guidelines [48], were conducted 3–4 h following capture, irrespective of abdominal (blood-fed) status. Larvae sampled from Ahero were raised to adults in the insectary following standard larval rearing methodologies [47]. Three-day-old adult, non-blood fed females raised from larvae were exposed to 0.75% permethrin-impregnated papers [48]. *Anopheles gambiae* (Kisumu Strain) permethrin-susceptible mosquitoes were used in paired controls. Negative controls using only carrier oil and susceptible controls were not conducted. For all specimens, knockdown rates were recorded at 10-min intervals for 1 h and mortality recorded 24 h post-exposure. Mosquitoes that remained alive after 24 h of exposure were killed at − 20 °C. All mosquitoes were labeled by assay phenotype (knocked down/alive) and morphologically identified to species before being individually preserved in Eppendorf tubes containing silica gel. Phenotypic insecticide resistance status was determined based on mortality rates according to WHO [48], where mortality rates between 98 and 100% indicated susceptibility, 90–97% suggested possible resistance that needed additional analysis and < 90% indicated resistance.

### Mosquito species identification

All adult *Anopheles* were morphologically identified using a dissecting microscope according to standard taxonomic keys [49]. A subset of morphologically identified secondary vector specimens were sequenced (ABI3730XL, Applied Biosystems, USA) at the ribosomal DNA internal transcribed spacer region 2 (ITS2) and/or cytochrome oxidase subunit 1 (*CO1*) loci towards species determination [18, 23, 25]. Specimens sequenced were randomly chosen across all adult trapping methods and sites. DNA was extracted from legs and wings. Samples were first sequenced at the ITS2 loci, and then a subset of samples with successful ITS2 sequences were also sequenced at the *CO1* loci. Molecular identification was conducted blind to morphological identity to prevent any bias in the analysis. Final species confirmation required high sequence identity (98% or greater) to voucher sequences in multiple databases. *CO1* and ITS2 database comparisons for each sample were paired to determine species when either *CO1* or ITS2 alone did not produce significant results to voucher sequences. Consensus sequences were manually inspected for insertions, deletions and repeat regions to ensure these sequence differences did not inflate divergence and decrease identity scores [18, 23, 25]. Consensus sequences of each sequence group were compared (BLASTn) to the NCBI nr and BOLD [50] databases to identify species. Molecularly identified *An. gambiae* (*s.l.*) samples were identified to species using a PCR diagnostic assay [51].

### Sporozoite infection

All female secondary vector specimens were dissected, and the thoraces and heads were analyzed for the presence of circum-sporozoite antigen of *P. falciparum* using an Enzyme-Linked Immuno-Sorbent Assay (ELISA) kit (MRA-890, MR4, ATCC, Manassas, VA). CS-ELISA-positive malaria vectors were determined using the ELISA kit procedure [52]. All sporozoite-positive mosquitoes were molecularly identified to species as above. The proportion positive for *P. falciparum* sporozoites was computed as the proportion of vectors ELISA positive for the CS protein out of the total analyzed, with the results presented as infection rates.

### Data analysis

Data collected on paper data collection sheets were entered into Microsoft Excel. Phenotypic insecticide resistance status was determined based on mortality rates according to WHO [48]—where mortality rates between 98 and 100% indicated susceptibility, 90%–97% suggested possible resistance with additional analysis required and < 90% indicated resistance. The sporozoite rate was computed as the proportion of vectors positive for circumsporozoite (CS) protein out of the sum total analyzed and the results presented as infection rates.

### Ethical clearance

This study was approved by the Ethical Review Board at the Kenya Medical Research Institute (KEMRI). Permission to carry out this study was obtained by the area chiefs and sub-chiefs. Informed consent for sampling of mosquito specimens was obtained from household and field owners.

## Results

Multiple collection methods were used over the rainy season to evaluating the species compositions, bionomic traits, proportion positive for *P. falciparum* sporozoites and insecticide resistance frequency of secondary *Anopheles* vectors (non-*An. gambiae* (*s.l.*) and non-*An. funestus*) in Kisumu County, western Kenya.

### Species composition and bionomics

The overall proportion of adult secondary malaria vectors sampled (61.43%; *n* = 887) was higher than that of primary vectors (38.57%; *n* = 557). Morphological identification of adult secondary vectors (*n* = 887) demonstrated the presence of *An. coustani* (57.0%, *n* = 506) and *An. pharoensis* (42.9%, *n* = 381) at both sites sampled (Kisian and Kakola-Ombaka) (Table 1).

**Table 1:** Morphologically identified adult *Anopheles* samples by site based on sampling method in Kisumu County

Site	Vector type	Primary vector				Secondary vectors			
	*Anopheles* species	*An. gambiae s.l*		*An. funestus s.l*		*An. coustani*		*An. pharoensis*	
	Sampling method	Total	Female/trap/night	Total	Female/trap/night	Total	Female/trap/night	Total	Female/trap/night
Kakola-Ombaka	CDC LT inside	51	0.06	34	0.04	65	0.08	28	0.03
	CDC-LT outside	51	0.06	8	0.01	108	0.13	113	0.13
	Animal-baited traps	85	1.01	59	0.70	307	3.65	204	2.43
Kisian	Animal-baited traps	183	2.18	86	1.02	26	0.31	36	0.43

Primary vectors sampled (*An. gambiae* (*s.l.*) and *An. funestus* (*s.l.*)) are also included to demonstrate the relevance of secondary species in context with the overall vectors present. Capture rates are standardized by female specimens per trap per night

**Fig. 2:**
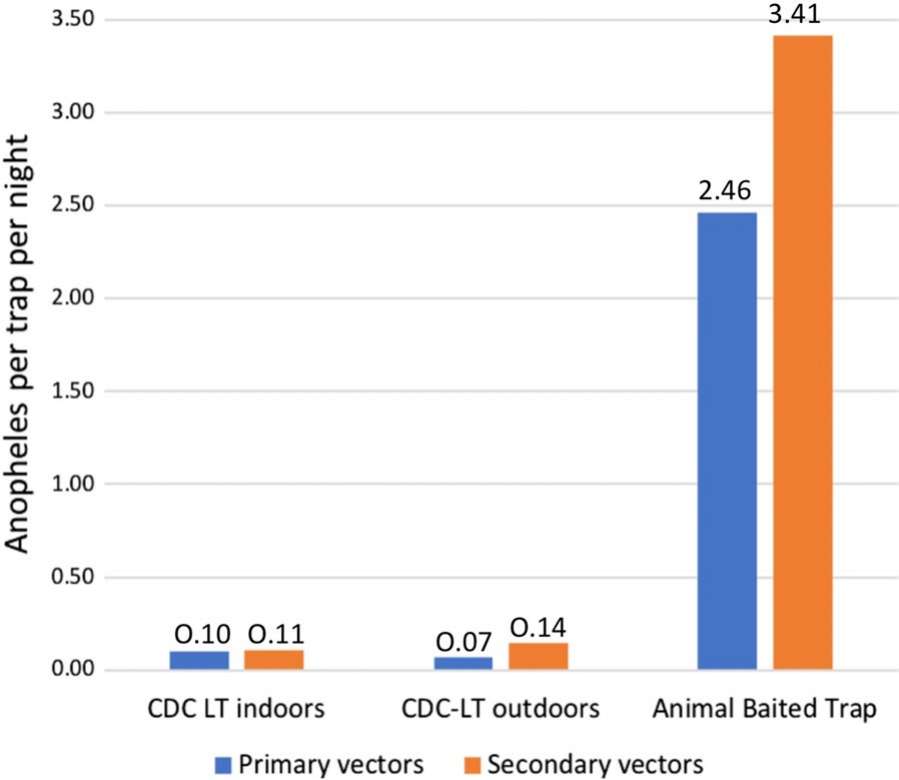
Standardized trapping densities demonstrate that indoor CDC-LTs captured almost equal numbers of primary and secondary vectors, outdoor CDC-LTs captured half the number of primary vectors, while animal-baited traps captured the most specimens, with more secondary vectors. *Anopheles* captured per trap per night are denoted above each bar

When compared to primary vectors and standardized to collection method (specimens per trap per night), indoor CDC-LTs captured equal numbers of primary and secondary vectors (0.10 and 0.11/trap/night, respectively) and outdoor CDC-LTs captured half the number of primary vectors (0.07 primary and 0.14 secondary/trap/night), while animal-baited traps captured the most primary vectors (2.46/trap/night) with more secondary vectors (3.41/trap/night) (Fig. 2). Overall, CDC-LTs captures were composed of 31.4% (*n* = 144) primary vectors and 68.5% (*n* = 314) secondary vectors, while ABT sampling had 41.9% (*n* = 413) primary vectors and 58.1% (*n* = 573) secondary vectors. Kisian was the only site with higher primary vectors in the ABT.

### Molecular identification of *Anopheles* species

The random subset of morphologically identified samples was successfully sequenced (*n* = 83) at the ITS2 and/or *CO1* regions for molecular species identification. Five species were identified molecularly (Table 2). Of the specimens morphologically identified as *An. pharoensis,* two were molecularly identified as *An. gambiae* (*s.l.*) and one as *An. christyi*. A single specimen, also morphologically misidentified as *An. pharoensis*, had its ITS2 sequence only 76% similar to that of *An. coustani* (henceforth called *An. cf. coustani*) and is possibly an unknown member of the *An. coustani* complex or group. The fourth species identified was *An. sp. 15 BSL-2014*—a potential member of the *An. coustani* group [23]—and was consequently identified as *An. coustani* morphologically. The last species—henceforth called *An. cf. pharoensis*—was primarily morphologically identified as *An. pharoensis* and was molecularly determined to be a member of Series Cellia, with an ITS2 sequence that matched that of *An. pharoensis,* while the *CO1* sequence was closest to that of *An. squamosus* (NCBI*)* and an unknown species—*An. KHH4* [53]. Approximately 77% (*n* = 64) of morphological identifications matched molecular identifications when including cryptic identifications. Species-specific ITS2 and *CO1* sequences may be accessed in GenBank (ITS2: MW578718 to MW578722; *CO1*: MW555792 to MW555794).

**Table 2:** Morphological versus molecular identifications

Morphological species identification	Molecular species identification				
	An. arabiensis*	*An. christyi*	*An. sp. 15 BSL-2014*	An. cf. pharoensis*	*An. cf. coustani*
*An. coustani s.l*	0	0	50	9	0
*An. pharoensis*	2	1	6	14	1
Total	2 (1)	1	56	23 (5)	1

Two secondary vectors were identified morphologically (*An. coustani* (*s.l.*) and *An. pharoensis)*, while five species were identified molecularly

Specimens that were found to be sporozoite positive by ELISA are labeled with an asterisk (*) with the number of positive samples in parentheses

### Sporozoite ELISAs

Of 791 mosquitoes tested for the presence of *P. falciparum* parasites, six tested positive for *Plasmodium* CS antigen. All six sporozoite-positive samples were identified molecularly for the most accurate species determination (Table 2).

Five positive samples were identified as *An. cf. pharoensis*. A single specimen of *An. arabiensis* (confirmed with PCR) [51] was also positive. All *Plasmodium*-positive secondary vector samples were from Kakola-Ombaka and were from outdoor collections—CDC-LTs (*n* = 2) as well as ABTs (*n* = 3). Since only a subset of samples was molecularly identified, species-specific infection rates were not possible. Overall, 5 of 789 (minus two molecularly identified *An. arabiensis* samples) secondary vectors tested were CS positive pointing to an overall secondary vector infection rate of 0.63%.

### Insecticide resistance

Insecticide resistance to permethrin was based on both morphologically identified adults captured as well as on larvae raised to adults (Table 3).

**Table 3:** Mortality rates from WHO bioassays based on morphological species, sampling methods and sites as well as adult and larval-based samples combined demonstrate susceptibility to permethrin

Site	Sampling method	Control mortality-*An. gambiae* (total tested)	Mortality-*An. coustani (s.l.)* (total tested)	Mortality-*An. pharoensis* (total tested)	Mortality-secondary vectors (total tested)
Kakola-Ombaka	CDC-LT inside	100% (93)	100% (65)	100% (28)	99.43% (887)
	CDC-LT outside	100% (221)	100% (108)	99.12% (113)	
	ABT	100% (511)	99.67% (307)	98.53% (204)	
Kisian	ABT	100% (62)	100% (26)	100% (36)	
Ahero	Larvae	100% (217)	98.3% (119)	100% (100)	99.10% (219)

Since each morphologically identified species was composed of multiple (molecularly identified) species, overall adult secondary vector mortality rates were 99.43% (*n* = 887). Larvae sampled from Ahero that were raised to adults also demonstrated high degrees of mortality (overall 99.10% mortality, *n* = 219). All adult and larval-raised specimens (whether based on morphological species identification, trap type or site) were classified as being susceptible to permethrin.

## Discussion

This study characterized the presence, bionomic traits, *P. falciparum* infected proportions and insecticide susceptibility status of secondary vector species from Kisian, Ahero and Kakola-Ombaka—sites across Kisumu County, Kenya—with several important outcomes.

Multiple collection methods that took advantage of multiple behaviors (endophily, exophily, anthropophily and zoophily) were used over the rainy season to ensure that as many vectors were sampled as possible. In addition to the primary vectors (*An. gambiae* (*s.l.*) and *An. funestus*), four other *Anopheles* species were identified molecularly that belonged to both identified species as well as cryptic or species complexes (Table 2). The known species was a single specimen of *An. christyi* identified by its ITS2 sequence (100% identical to that of *An. christyi*). *Anopheles sp. 15 BSL-2014,* identified from both ITS2 (100% identical) and *CO1* (99% identical) sequences, represented the majority of the samples sequenced (67.4%), is part of the *An. coustani* complex and was first detected in the Kenyan highlands. This species is likely a close relative of *An. coustani*; however, low sequence similarity to Group Coustani sequences in the database (such as *An. paludis*) and the lack of identifying sequences from species such as *An. crypticus* and *An. caliginosus* render further granularity with molecular identification impossible.

Another species, identified from a single specimen, had only partial overlap (76%) with the *An. coustani* ITS2 sequence, and of this overlap, there was low (79%) similarity. Other much lower similarity scores with this sequence demonstrated similarity to *An. pullus* and *An. yatsushiroensis*, part of the Group Coustani sibling—Group Hyrcanus, both part of Series Myzorhynchus. The closer similarity to *An. coustani* suggests that this unknown species may be part of the *An. coustani* complex. The majority of specimens of the fourth species demonstrated high similarity (98%) to the ITS2 sequence from *An. pharoensis*, with *CO1* sequences being most similar (97%) to *An. squamosus* and a novel species *An. KHH4* [53]. *Anopheles pharoensis* is believed to consist of two species—*An. argenteolobatus* and *An. cydippis,* based on polytene chromosome banding patterns [54]. However, the lack of *An. argenteolobatus* and/or *An. cydippis* ITS2 or *CO1* sequences in the reference databases precludes this determination.

A DNA barcoding study [53] utilizing *CO1* for species identification revealed the presence of *An. KHH4* in Western Kenya with a phylogenetic analysis demonstrating its close relationship with *An. squamosus.* This is the first time an ITS2 sequence has been paired with this *CO1* sequence. These sequences may possibly document one of the two species identified within the taxon *An. pharoensis* Theobald [54].

Morphological identification techniques have their limitations as seen in this study. Here *An. gambiae* (*s.l.*) and *An. christyi* specimens were misidentified as *An. pharoensis* and four novel species were identified as two species based on morphological characteristics. The difference seen between molecular and morphological results here points to the lack of data on secondary vectors in western Kenya. Secondary vectors are either not utilized for primary analysis or identified using morphological keys leaving out the possibility of the detection of species complexes and novel species as seen in other studies utilizing molecular methods [18, 23–25]. The association of specific species with their bionomic traits—especially those that impact intervention efficacy, such as biting spaces and times and insecticide resistance—are vital when understanding where and when transmission is occurring as well as species-specific impacts of intervention strategies. The use of molecular tools, alongside morphological identifications, as is routinely done for the *An. gambiae* complex and the *An. funestus* group, would ensure accurate and precise characterization of malaria vectors when characterizing vector-based transmission systems. It is important to note that the molecular analysis represents only 9.4% (83 of 887) of the adult *Anopheles* specimens captured here, and a more comprehensive molecular analysis may reveal the presence of other species.

The unlikely morphological misidentification of *An. coustani* as *An. pharoensis* could not be resolved because the specimens were used for ELISA and/or DNA extractions. This demonstrates the need to retain voucher specimens especially when unidentifiable specimens are found. These voucher specimens serve as referencing entities, recording and detailing the identity of samples used in a study, and may clarify misidentifications and novel specimens in cases such as this. A single leg or a few scales may be utilized for molecular identification with the morphological integrity retained for any further required data validation. These data may also point to and advocate for additional training in morphological identifications.

Rare, unknown and novel species are common in the *Anopheles* genus and have been reported across the world [18, 23, 25, 55, 56]. More interestingly, some of these species were found to be susceptible to *P. falciparum* infection. Undescribed species have previously been reported in the Western region of Kenya with some being infected with sporozoites [8, 23, 57]. Further studies are required to determine their vector status, relationship to other species as well as bionomic characteristics that relate to transmission and intervention efficacy. Climate change and local ecological niches are among the factors that may play a role in the occurrence and spread of these novel species [24]. The ability of these species to harbor *Plasmodium* parasites suggests their role in malaria transmission and the need for them to be targeted by vector control tools.

This study not only demonstrates the unexpected species composition of secondary vectors but also argues that their ‘secondary’ contributions to transmission may be more than expected. When comparing standardized trapping rates (Table 1) 0.10 and 0.07 primary vectors (*An. gambiae* (*s.l.*) and *An. funestus* (*s.l.*) together) were caught per trap per night by CDC-LTs indoors and outdoors respectively—pointing to their endophilic nature. However, higher trapping rates—0.11 and 0.26/trap/night indoors and outdoors respectively—of secondary vectors were sampled at the same time pointing to both exophilic behaviors as well as larger possible population sizes. Compared to a study [7] done in western Kenya, this study showed a significant increase in secondary vectors collected. This may be attributed to extensive use of ITNs in the region leading to diminished primary vectors and thus reduced competition for secondary vectors—hence their increased density.

When looking at ABTs, primary vector capture rates were 1.71/ABT/night while that of secondary vectors was 6.08/trap per night in Kakola-Ombaka suggesting both zoophily and greater population sizes. This was however reversed in Kisian with 3.20 primary vectors captured per ABT per night while only 0.73 secondary vectors were sampled per trap per night at the same time. With Kisian having a high density of domestic cattle, data may reflect species-specific compositions of the secondary vector species with some possibly being more attracted to bovines than others. Since only a small proportion of secondary vectors was analyzed molecularly, this could not be clarified. Overall, these comparisons suggest that secondary vector biting rates may be equivalent to those of primary vectors (based on CDC-LT catches) while outdoor biting rates may be significantly more (up to 3.7 × more).

Overall secondary vectors were the majority of the collections composing 68.6% of CDC-LT catches and 58.1% of ABT collections. These results are unexpectedly high when compared to other studies [7, 28, 45]. This may reflect the multiple trapping methods used in this study as well as changes in these vector densities over time – reductions in primary vectors with indoor interventions [28] alongside escalations of secondary vector densities with reduced competition [58, 59].

Sporozoite-positive proportions of 0.63% seen in the secondary vectors sampled indicate a large contribution to transmission since biting rates are possibly equivalent to (indoors) or more than (outdoors) those of primary vectors [9]. All sporozoite-positive samples were also captured outdoors (outdoor CDC-LT: *n* = 2; ABT: *n* = 3) further pointing to possibly more transmission being outdoors with these vectors. Establishment of sporozoite infection among these secondary malaria vectors is in accordance with other studies [12, 16]. The overall *P. falciparum* infection rate was low and this could be attributed to very minimal vector-human contact driven by exophily and efficacy of insecticides [60]. Secondary vectors with exophilic and zoophilic tendencies—or rather higher acceptance rates of alternative blood meal sources when human hosts are unavailable—may help maintain low but constant transmission [21, 61–63]. When factoring molecular species identifications, and the possibility that some *Anopheles* species identified here may not be vectors, species-specific sporozoite rates would be greater than the overall rate reported here.

Phenotypic bioassays demonstrate the susceptibility of these secondary vectors to permethrin (Table 3). The use of both wild-caught adult females without regard to abdominal (blood-fed) status and those raised from larvae enabled the determination of baseline genetic resistance (from unexposed and non-blood fed larvae) as well as resistance of adults in the field factoring in possible prior exposure as well as various physiological states. Though physiological characteristics such as age and blood feeding status can confer increased tolerance to insecticides [64], the lack of insecticide resistance seen in both sets of vectors suggests insufficient selection processes due to less exposure to indoor insecticide-based interventions (LLINs and IRS) because of both exophily and zoophily. However, their present abundance and the increased focus on the incorporation of outdoor vector control tools [65] pose a risk of pyrethroid-based control failure—were these populations to develop resistance. It is therefore vital to continue monitoring their susceptibility status since pyrethroid insecticides are still in use in public health and agriculture. The worst-case scenario is where widespread resistance to all four classes of insecticides currently in use is seen in primary vectors [6, 67]. The very low levels of survival in this study (< 1%) still point to the possibility of ongoing low levels of selection. A study that evaluates the presence of any underlying genetic markers of resistance would serve as an early warning signal of insecticide resistance before it is expressed phenotypically and may impact intervention efficacy [68]. Further insecticide resistance evaluations in conjunction with molecular identifications will further enable species-specific insecticide resistance characterizations.

Since the terms ‘primary’ and ‘secondary’ are arbitrarily assigned to vectors based on characterized contributions to transmission at that point in time, changes in species compositions, insecticide pressure, climate change, changes in land use, etc., may influence the contributions of specific species to malaria transmission. For instance, in the 1950s *An. rivulorum* and *An. parensis* replaced *An. funestus* as the primary vector in Kenya and Tanzania [58]—the density of *An. rivulorum* increased almost seven times with a concurrent reduction of *An. gambiae* (*s.l.*). More recently, the lowland areas of Western Kenya have reported an increase in the density of *An. rivolurum* [16] attributed to reduced interspecific competition caused by current vector control interventions diminishing populations of primary vectors. This paradigm is reflected in this study where both population sizes (Table 1) and sporozoite rates of ‘secondary’ vectors are equivalent to or more than those of ‘primary’ vectors—suggesting the greater role of these vectors in transmission and the possible elevation to primary vector status based on further studies.

This small study with important outcomes suggests the need for more comprehensive collections of secondary vectors in parallel with molecular species identifications. This comprehensive analysis would include more sites and specimens for greater understanding of species compositions and their bionomics—including insecticide resistance. Collections over multiple seasons would enable temporal evaluations of these vectors and the drivers of their populations.

## Conclusions

Vectors characterized as ‘secondary’ in Kisumu County are of greater potential significance to transmission as demonstrated by their higher densities and presence of sporozoites. Their exophilic and zoophilic natures indicate that they evade indoor interventions and may explain the lack of phenotypic resistance. These traits combined with their densities may amplify outdoor and residual transmission. This study indicates that the possible primary role of these ‘secondary’ vectors in malaria transmission may be the result of the apparent failure of current interventions targeting primary vectors to achieve complete malaria control. Here, local transmission dynamics may elevate the contributions of ‘secondary vectors’ to primary status—pointing to the need for routine surveillance to capture these important drivers of transmission. Further studies are required to understand the temporal species compositions and bionomics of these vectors in relation to malaria transmission in western Kenya.

## Data Availability

The original data applied and/or evaluated throughout the current project are accessible from the corresponding author on judicious appeal or from Kenyatta University Digital Library.
